# In-vivo assessment of the diagnostic performance of DENSE in patients with myocardial infarction

**DOI:** 10.1186/1532-429X-16-S1-P190

**Published:** 2014-01-16

**Authors:** Christie McComb, David Carrick, Rosemary Woodward, John D McClure, Aleksandra Radjenovic, Colin Berry, John Foster

**Affiliations:** 1 BHF Glasgow Cardiovascular Research Centre, Glasgow, UK; 2 Clinical Physics, NHS Greater Glasgow and Clyde, Glasgow, UK; 3 Cardiology, Golden Jubilee National Hospital, Glasgow, UK; 4 MRI, Golden Jubilee National Hospital, Glasgow, UK

## Background

In patients with myocardial infarction (MI), an important factor in determining the long-term prognosis is the degree of regional contractile dysfunction. DENSE (Displacement ENcoding with Stimulated Echoes) is a technique which allows quantification of myocardial strain[1], and which has been shown to be more sensitive to the presence of late gadolinium enhancement (LGE) than wall thickening measured from cine images[2]. The aim of this study was to further investigate the performance of DENSE for the diagnosis and assessment of myocardial infarction.

## Methods

50 male patients (age 56 ± 10 years) within 7 days of MI and 30 healthy male controls (age 45 ± 18 years) underwent CMR on a 1.5T Siemens Avanto. The protocol included DENSE and LGE (patients only) obtained from a single mid-ventricular short-axis slice, which was divided into 6 segments for analysis. The percentage of each segment which contained LGE was calculated using a threshold of mean+5SD of remote myocardium intensity. DENSE images were analysed to obtain a value for peak circumferential strain (Ecc). The following analyses were performed: (i) intra- and inter-operator and inter-scan repeatability, (ii) sensitivity and specificity for the detection of LGE, using a reference range established from control data, and ROC analysis and (iii) the ability to distinguish between non-infarcted, <50% and >50% infarction, and between remote, adjacent and infarcted segments.

## Results

**Figure 1:**
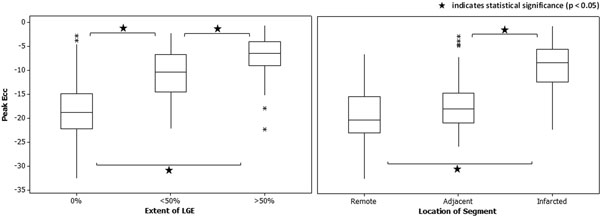


The results of (i) repeatability and (ii) sensitivity, specificity and area under curve (AUC) are summarised in Table 1, along with the percentages of segments correctly identified when grouped according to extent of LGE. The reference range was calculated to be (-11.8, -27.0). Inter-operator repeatability was assessed using Levene's test (variance) and a paired t-test (mean), and no statistically significant differences were found. A paired t-test found no statistically significant difference for inter-scan repeatability. The results of (iii) are illustrated in Figure 1. Comparisons between categories were performed using a one-way ANOVA with Tukey's post-hoc test.

**Table 1:** 

Test	Result
Intra-operator repeatability (CoV %)	10

Inter-operator limits of agreement	(-3.1, 2.9)

Inter-scan limits of agreement	(-1.8, 2.0)

Sensitivity (%)	71

Specificity (%)	86

AUC	0.87

Segments correctly identified (%):	

1 - 25% LGE	59

26 - 50% LGE	70

51 - 75% LGE	77

76 - 100% LGE	93

## Conclusions

DENSE can be successfully applied in a clinical setting, and provides repeatable results. The sensitivity and specificity of the technique for detecting the presence of LGE are good, and the number of segments with LGE correctly identified increases as the extent of LGE increases. Peak Ecc can be used to distinguish between non-infarcted, <50% and >50% infarcted segments, but cannot identify segments which are adjacent to infarction.

## Funding

N/A.
